# Micro-segmental hair analysis: detailed procedures and applications in forensic toxicology

**DOI:** 10.1007/s11419-022-00619-9

**Published:** 2022-03-21

**Authors:** Kenji Kuwayama, Hajime Miyaguchi, Tatsuyuki Kanamori, Kenji Tsujikawa, Tadashi Yamamuro, Hiroki Segawa, Yuki Okada, Yuko T. Iwata

**Affiliations:** National Research Institute of Police Science, 6-3-1 Kashiwanoha, Kashiwa, Chiba 277-0882 Japan

**Keywords:** Hair analysis, Micro-segmental analysis, Drug-facilitated crimes (DFCs), Single hair strand, Estimation of the day of drug consumption, LC–MS/MS

## Abstract

**Purpose:**

Since the 1980s, the detection sensitivity of mass spectrometers has increased by improving the analysis of drugs in hair. Accordingly, the number of hair strands required for the analysis has decreased. The length of the hair segment used in the analysis has also shortened. In 2016, micro-segmental hair analysis (MSA), which cuts a single hair strand at a 0.4-mm interval corresponding to a hair growth length of approximately one day, was developed. The advantage of MSA is that the analytical results provide powerful evidence of drug use in the investigation of drug-related crimes and detailed information about the mechanism of drug uptake into hair. This review article focuses on the MSA technique and its applications in forensic toxicology.

**Methods:**

Multiple databases, such as SciFinder, PubMed, and Google, were utilized to collect relevant reports referring to MSA and drug analysis in hair. The experiences of our research group on the MSA were also included in this review.

**Results:**

The analytical results provide a detailed drug distribution profile in a hair strand, which is useful for examining the mechanism of drug uptake into hair in detail. Additionally, the analytical method has been used for various scenarios in forensic toxicology, such as the estimation of days of drug consumption and death.

**Conclusions:**

The detailed procedures are summarized so that beginners can use the analytical method in their laboratories. Moreover, some application examples are presented, and the limitations of the current analytical method and future perspectives are described.

## Introduction

Analytical methods for the detection of drugs in hair have been improved greatly with the development of analytical instruments. Table 1 shows some pioneering studies that made breakthroughs in the analysis of drugs in human hair.

**Table 1:** History of drug analysis in human hair

Breakthrough	Sample type (amount)	Segment length	Analyte	Detection (LOD)	Reference(s)
Dawn of drug analysis in human hair	Bulk (10 mg)	Whole hair, proximal and distal 2.5-cm segments	Opiates	RIA (1–10 ng)	Baumgartner et al. (1979) [1]
Single hair analysis	Single hair (0.4 mg)	Whole hair	Phenobarbital	RIA (1.25 ng)	Smith et al. (1981) [2]
Use of GC–MS	Bulk (5.9–227 mg)	Whole hair	Methamphetamine, amphetamine, antidepressants, nicotine	GC and GC–MS	Ishiyama et al. (1983) [3]
	Bulk (50–100 mg)	Whole hair	Chloroquine, desethylchloroquine	GC–MS	Viala et al. (1983) [4]
Sub-ng detection	Single hair	Whole hair	Methamphetamine, amphetamine	GC–MS (10 pg)	Suzuki et al. (1984) [5]
Use of LC	Bulk (100–200 mg)	Whole hair (4 cm)	Morphine	LC (60 pg)	Marigo et al. (1986) [6]
Single 1-cm segmental analysis	Single hair	1-cm Interval	Haloperidol	LC	Matsuno et al. (1990) [12]
Sub-cm segmental analysis	Bulk (5 strands) and single hair	2.5-mm Interval	Quinolone derivative	LC	Uematsu et al. (1993) [13]
Use of LC–MS	Bulk (40 mg)	Whole hair	Buprenorphine	LC–MS (200 pg)	Tracqui et al. (1997) [7]
	Bulk (60 mg)	Whole hair	Methadone	LC–MS	Kintz et al. (1997) [8]
Use of MS imaging	Single hair	Laser spot at 30-μm step	Methamphetamine	MALDI-MS imaging	Miki et al. (2011) [9, 10]
	Single hair	Laser spot at 1-mm step	Cocaine	MALDI-MS imaging	Porta et al. (2011) [11]
Segmentation (1 mm)	Single hair	Interval (1 mm)	Methoxyphenamine	LC–MS/MS (100 fg)	Kamata et al. (2015) [14]
Sub-mm segmentation	Single hair	Interval (0.4 mm)	Chlorpheniramine	LC–MS/MS (4 fg)	Kuwayama et al. (2016) [15]
Use of ITM	Single hair	Interval (0.4 mm)	Cold medicines, hay fever medicines	LC–MS/MS (2–40 fg)	Kuwayama et al. (2018) [17]

*LOD* limit of detection, *RIA* radioimmunoassay, *GC* gas chromatography, *MS* mass spectrometry, *LC* liquid chromatography, *MALDI* matrix-assisted laser desorption/ionization, *ITM* internal temporal marker

First, we look back on the history of drug analysis in hair in terms of analytical instruments used. In 1979, Baumgartner et al. [1] first reported analytical results for opiates in human hair. They used a radioimmunoassay to sensitively detect analytes using a 10-mg hair sample. A radioimmunoassay analysis using just a single hair was reported by Smith et al. in 1981 [2]. The mass of hair used for the analysis was only 0.4 mg. Meanwhile, drug detection using gas chromatography (GC)–mass spectrometry (MS) was developed in 1983 [3, 4]. The use of MS has made a significant contribution to the reliable identification of compounds. Suzuki et al. [5] succeeded in achieving sub-nanogram drug detection from a single hair using GC–MS with chemical ionization in 1984. Subsequently, liquid chromatography (LC) has also been used for drug analysis in hair [6]. Since the late 1990s, LC–MS has become a major analytical approach for drug analysis owing to high detection sensitivity for most drugs and easy usability [7, 8]. Advances in the performance of MS, such as tandem MS, high sensitivity, and high resolution, have been made one after another and applied to drug analysis in hair. Contemporarily, such highly sensitive and selective analysis has enabled the identification and quantification of trace amounts of drugs in small amounts of hair. Imaging of drugs on a hair strand using matrix-assisted laser desorption/ionization (MALDI)-MS, first reported in 2011, has brought another new approach to drug analysis in hair [9–11].

Next, we look back on the history in terms of the length of hair segment used. Segmental hair analysis at intervals of several centimeters has been performed since the early history of hair analysis. In 1979, Baumgartner et al. [1] separately analyzed proximal and distal 2.5-cm hair segments. They showed that the drug concentration differed between hair regions, reflecting the time of drug use. In 1990, Matsuno et al. [12] reported the distribution of haloperidol in a hair strand by segmenting a single hair strand at 1-cm intervals. Moreover, their group also realized a 2.5-mm segmentation and showed that the detailed distribution measurement along hair length could serve as a temporal marker and be used to estimate hair growth rate [13]. Kamata et al. [14] measured the distribution of methoxyphenamine in a single hair at 1-mm intervals using LC–MS/MS and using LC–MS/MS together with MALDI-MS imaging, and went on to show that there were two major drug incorporation sites. In 2016, Kuwayama et al. [15] developed a method to measure drug distributions in a single hair strand by segmenting a hair strand at 0.4-mm interval, corresponding to a hair growth length of approximately one day. The method was termed “micro-segmental hair analysis (MSA)” as a 0.4-mm segment weighs several micrograms [16]. The analytical results provided a detailed drug distribution profile in a hair strand, which is useful for examining the mechanism of drug uptake into hair in detail. Additionally, the analytical method has been used for various scenarios in forensic toxicology, such as the estimation of days of drug consumption and death. Moreover, their group proposed a procedure to accurately estimate the day of drug intake using internal temporal markers (ITMs), as described below in detail [17].

The aim of this review is to foster the spread of MSA and its development in many forensic laboratories by explaining the detailed procedures and providing examples of practical application.

## Literature search

Reports referring to drug analysis in human hair were collected using the keywords “drug” and “hair” in multiple databases, such as SciFinder, PubMed, and Google. The full text was carefully checked according to their requirements.

## Analytical methods for drug detection in hair

### Usage amount of hair sample

In practical hair analysis in forensic toxicology, an analytical method is chosen depending upon the specific aims of the analysis (screening, proof of drug use, supporting data for drug detection from other specimens, estimation of drug-use history, etc.), the amount/number of hairs available, and detection sensitivity of the instruments used. There are several classifications in terms of the starting hair samples used.

One simple classification is bulk- or single-hair analysis. Bulk hair typically consists of tens of strands per sample [1, 3, 4, 6–8, 13]. The advantages of bulk-hair analysis are that it averages the growth rate and drug concentration between individual hair strands and increases the absolute amounts of analytes introduced into the instruments, thereby increasing the chances of detecting small but relevant quantities. By contrast, single-hair analysis enables information of drug use to be obtained from the minimum number of hair samples [2, 5, 9–17]. It should be noted that the analytical result from a single hair strand reflects the growth rate and drug amount in the analyzed individual hair strands. Therefore, several individual hair strands must be analyzed separately to comprehensively interpret the analytical results.

A further classification is whole-hair or segmental hair analysis. Whole hair contains information on drug use for the period from the previous habitual haircut to the sampling of hair for research [1–8]. The purpose of whole-hair analysis is to introduce sufficient analytes into analytical instruments using a limited number of hair strands. Whole-hair analysis may be sufficient to determine whether hair contains particular analytes. However, whole-hair analysis does not capture information about the duration of drug use. Additionally, the number of hair strands used for whole-hair analysis can vary depending on the length of individual hair strands. As the detection sensitivity of instruments improves, segmental hair analysis is recommended over whole-hair analysis, even when temporal information is unnecessary. A number of hair strands segmented at a regular length should always be analyzed to evaluate the analytical results in a fixed condition.

### Conventional segmental hair analysis

**Fig. 1:**
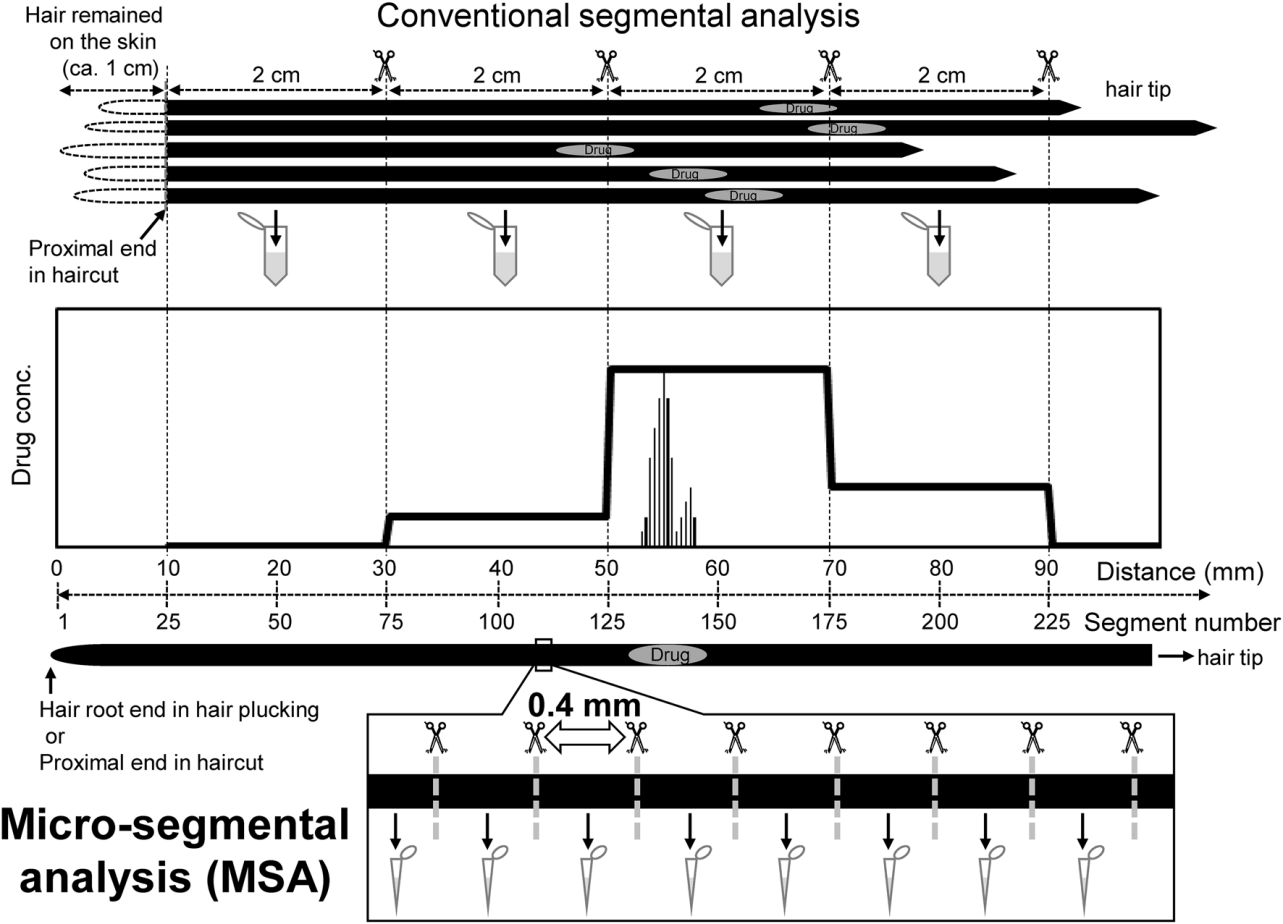
Differences of drug distribution profiles in hair between conventional segmental and micro-segmental analyses. Drug distribution curves in hair strands, collected several months after a participant consumed  a single dose of the drug, are drawn as examples. For conventional segmental analysis, multiple hair strands were cut near the scalp, and five hair strands were segmented at 2-cm interval from the proximal end. For micro-segmental analysis, a single hair strand with the root was plucked from the scalp and segmented at 0.4-mm interval from the root end

In conventional segmental analysis of bulk hair samples [13, 18–20], a lock of hair which is cut simultaneously from one site or multiple hair strands collected from some sites are used. Hair strands are arranged in parallel such that the proximal cut ends are lined up as the start line and then segmented at a regular length (Fig. 1). The segment length is determined based on the required time resolution. Generally, an interval of 1 cm or more is selected. When hair strands are cut at an interval of 2 cm, the drug-use history can be estimated at a temporal resolution of approximately 2 months, assuming that hair strands grow at an average rate of 1 cm per month [21, 22]. However, individual hair strands grow independently at different rates. Additionally, the length of hair strands remaining on the skin (distance from the hair root end to the proximal end in haircut) is also different among individual hair strands, although hair strands are usually cut near the scalp with scissors. Therefore, the distance from the proximal cut end to the drug-containing region does not completely match for all of the hair strands. Consequently, the distribution profile must be drawn as a wide bar graph, even if a single dose of the drug is ingested. It is useless to segment at intervals shorter than 1 cm in bulk-hair analysis because the differences in distribution between individual hair strands are averaged. Nevertheless, segmentation of a single hair at shorter intervals is useful for obtaining detailed drug distributions.

### Micro-segmental hair analysis (MSA)

In MSA, a single strand of hair, that is, a single fiber of hair is used. A hair strand is cut at sub-millimeter intervals, and each segment analyzed independently (Fig. 1) [15–17, 23–28]. The distribution profile shows a smooth curve as compared to that in the conventional segmental analysis. When a hair strand is segmented at a 0.4-mm interval, the distribution can reflect the drug-use history at the single-day level, assuming the hair strand grows at an average rate of 0.4 mm/day constantly. Because the number of hair strands used in MSA is much less than that in bulk hair analysis, hair strands with the root end can be plucked from donors from whom informed consent was obtained. Therefore, MSA is particularly useful for examining drug distribution around the hair root end [29].

**Fig. 2:**
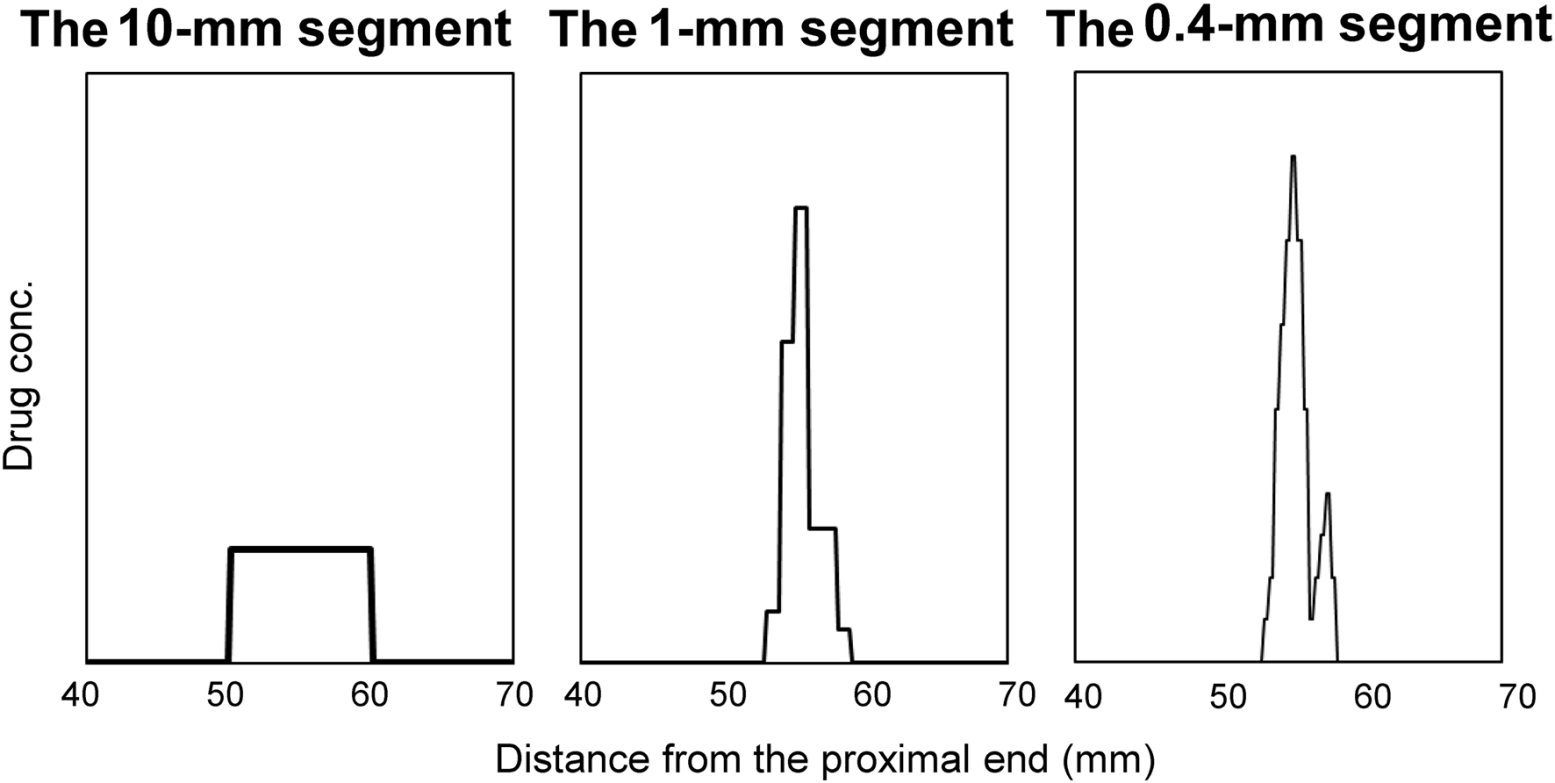
Differences of distribution curves depending on segment lengths. Drug distribution curves in hair strands segmented at 10 mm, 1 mm, and 0.4 mm intervals were simulated. The data around the drug-containing hair region shown in Fig. 1 were used. The simulation conditions were as follows: the drug in each segment was extracted using each  volume of extraction solution in proportion to the length (for example, 1 mL, 100 μL, and 40 μL, respectively), and the extraction rates and matrix effects depending on segment length were not changed

MSA is advantageous in terms of both detection sensitivity and spatial resolution. When a single dose of a drug is consumed, the drug is distributed in a specific region of individual hair strands. Of course, as the segment length decreases, the absolute amount of analyte in a segment decreases. However, the analyte can be extracted using a smaller volume of extraction solution. Additionally, matrix effects can be reduced because shorter segments contain fewer hair components (matrices) [26]. If the volume of extraction solution decreases in proportion to the segment length, the peak concentrations of analyte in a hair segment increase as the segment length is shorter (Fig. 2). Thus, the use of short segments can contribute to highly sensitive identification and quantification of analytes in hair.

## MSA procedure

### Hair collection

In the conventional hair analysis, 100 scalp hair strands are typically cut from the posterior vertex region of the heads of suspects and victims as specimens for the investigation of drug-related crimes [30]. If MSA is planned to be performed after a specific drug is detected using the conventional hair analysis, a portion (approximately five strands) of the hair collected should be left for MSA. If MSA is only performed, because the target drug has already been known, any haircut or hair plucking can be selected according to the donor’s preference. Haircut is painless but must be postponed until a target hair region comes above the scalp (at least 1 month after the occurrence of drug-related crime). However, hair plucking can be performed immediately after an incident. If the donor allows hair plucking, approximately five hair strands with the hair bulbs are plucked carefully from the scalp. Only hair strands that induce pain when plucked may be collected as specimens for drug testing. If a hair strand is plucked easily without pain, the strand must be excluded because there is a high probability that it is in the catagen or telogen phase [21, 22], growth is arrested and it will not reflect recent substance consumption.

### Preliminary examination for micro-segmentation of a single hair strand.

**Fig. 3:**
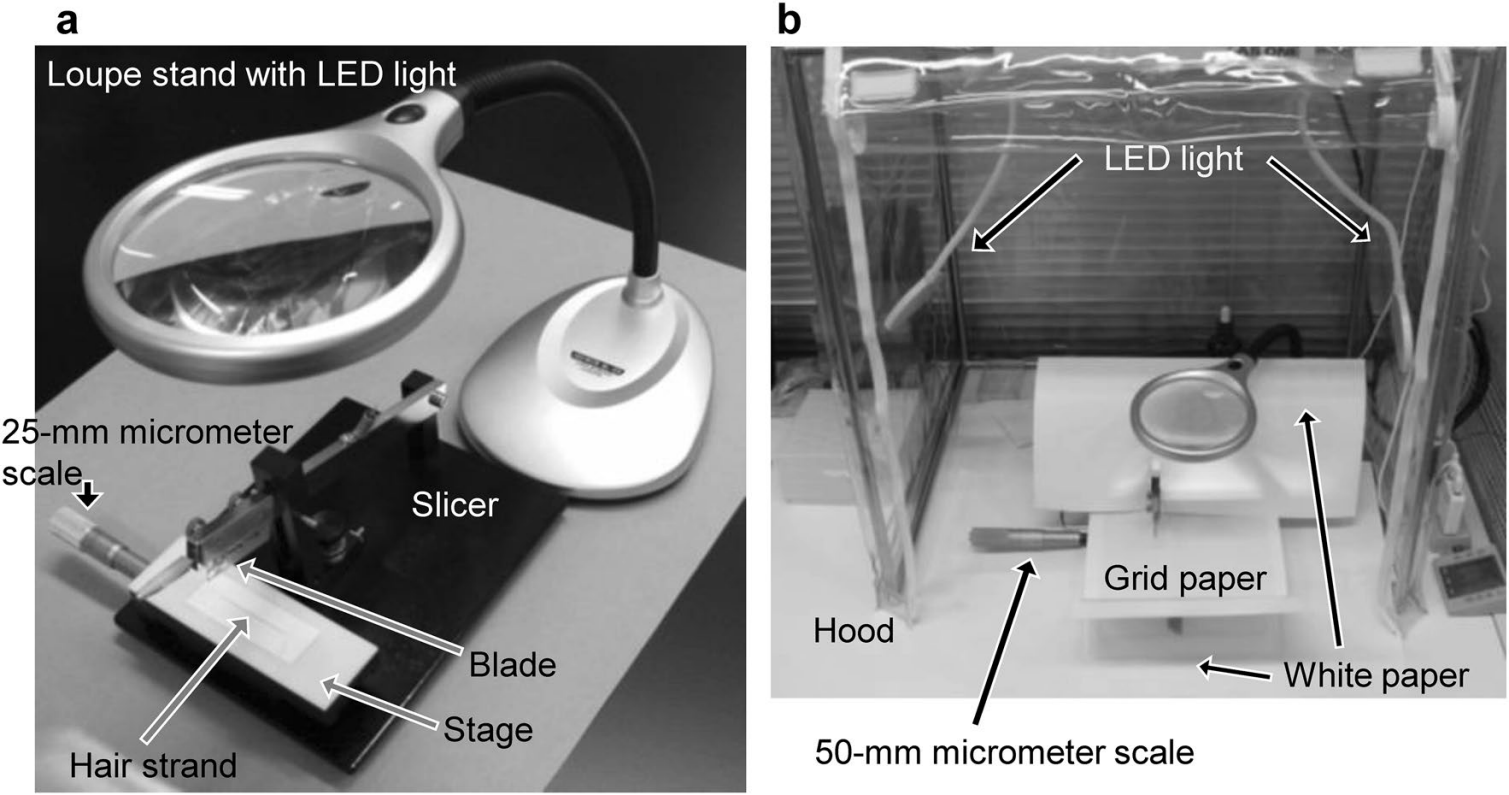
Tools and instruments used for micro-segmentation of a hair strand. **a** fundamental components, **b** optimized workspace

When the segment length is more than 1 mm, a hair strand is fixed on grid paper ruled into 1-mm squares with double-sided adhesive tape, and cut at a determined interval manually using a box cutter. To cut a hair strand to less than 1 mm precisely, tools like a slicer, micrometer scale, and magnifying glass are necessary. Kuwayama et al. [15, 31, 32] applied a tissue slicer to slice an organ block (Tissue slicer, 51425, Stoelting Co., Wood Dale, IL, USA) for hair segmentation. Figure 3a shows the fundamental tools used for micro-segmentation. The tissue slicer consists of a stage of width 12 cm × depth 4 cm, which is movable in the width direction using a 25-mm scale, and a guillotine cutter to go down vertically onto the stage. Because the micrometer scale has a resolution of 1 µm, a hair strand fixed on the stage can be cut easily and precisely at 0.4-mm (400 µm) interval. When the length of individual 0.4-mm segments was measured under a microscope with a micrometer, it was 0.403 ± 0.00419 mm (average ± standard deviation, *n* = 15 [16]). However, it is difficult to pick up tiny segments using tweezers. Indeed, a segment sometimes vanishes from sight during transfer into a microtube as the metallic luster of tweezers makes it difficult to see the segment. Sticking a segment on a tapered cotton swab is easier than picking it up with tweezers because adhesives derived from double-sided adhesive tape adsorbed on a segment surface help a segment stick onto the swab. Additionally, a segment on a cotton swab is easy to see because of the favorable color contrast between the dark hair segment and white cotton swab. Consequently, hair segmentation using a tissue slicer and collection of a segment using a cotton swab has been adopted for micro-segmentation in our laboratories.

### Optimized segmentation procedures

First, a single hair strand is weighed, the entire length measured, and the hair strands washed in the same manner as in the conventional analysis [30]. The weight of a segment is calculated by dividing the total weight by the number of segments in the entire length, assuming that the density and diameter are constant along the hair shaft because it is difficult to precisely measure the weight of each segment.

**Fig. 4:**
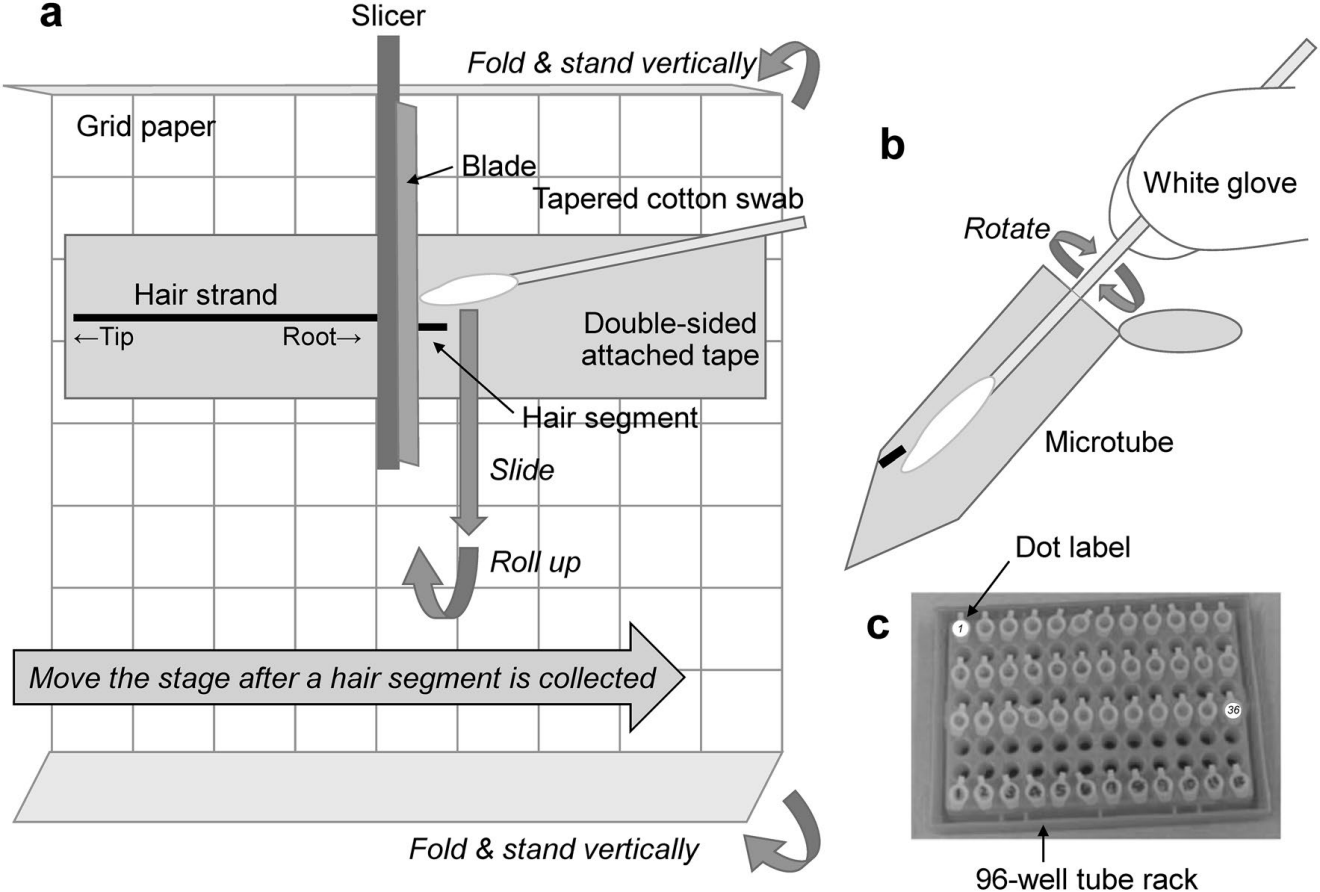
Procedures to collect micro-segments of a hair strand. **a** Picking of a hair segment on grid paper; **b** enclosure of a hair segment into a tube; **c** arrangement of tubes on a 96-well tube rack

Second, grid paper is attached to the stage of a tissue slicer equipped with a micrometer scale so that the vertical line of the grid paper is parallel to the blade (Fig. 4a). A piece of double-sided adhesive tape is attached along the horizontal lines of the grid paper. A single strand is placed stretching straight along a horizontal line on the tape with tweezers and pressed on to the tape firmly with a finger wearing a disposable finger cot. A white glove is worn on the hand, grasping a cotton swab to make it easy to see a segment. The stage position is adjusted using a micrometer scale so that the proximal hair end is immediately below the blade.

Third, the stage is moved 0.4 mm along the growth direction. A 0.4-mm segment is obtained by downing the blade. The micro-segment is placed in a microtube using a tapered cotton swab while observing the segment under a magnifying glass to avoid losing it (Fig. 4b). The series of procedures for stage movement, haircut, and segment collection are repeated until the targeted hair region is segmented or the stage position reaches the range limit of the micrometer scale. The blade is wiped with a cotton swab soaked in methanol to remove contaminants between every hair strand, segment or micro-segment.

### Tips for micro-segmentation

Typical tools used in MSA are summarized in Table 2. When the stage position reaches the range limit of the micrometer scale, the stage and proximal end of the residual hair strand must be returned to the starting positions. To minimize complications of the procedure, replacement of the default micrometer scale (25 mm) by a micrometer head with a longer travel range (50 mm) is recommended.

**Table 2:** Tools and instruments used for micro-segmental hair analysis

Tool/Instrument	Purpose of use	Recommended specification	Commercial product example
For segmentation			
Slicer	To cut a hair strand in round slices	A blade goes down to a stage vertically	Tissue slicer, 51,425 (Stoelting Co., Wood Dale, IL USA)
Stage	To place a hair strand	As large as possible to place a long hair strand straight Movable using a micrometer scale	Equipped to above slicer
Micrometer scale	To move a stage at a determined distance	Resolutions: 0.001 mm, 50mm or longer travel distance	A 25-mm micrometer head is equipped to above slicer Micrometer head, 50 mm travel distance, MHH2-50 T (Mitutoyo Corporation, Kawasaki, Japan)
Razor blade	To cut a hair strand	Stainless steel, single edge, ca. 0.2 mm thick (0.1-mm thick blade is not recommended because it bends easily)	Shaving blade, FHS (FEATHER Safety Razor Co. Ltd., Osaka, Japan)
Grid paper	To place a hair strand at right angle to a razor blade	Whitish background, light-colored lines ruled into 1-mm squares, A4 size or larger	Section paper, Ho-19 N (KOKUYO Co. Ltd., Osaka, Japan)
Double-sided adhesive tape	To fix a hair strand	Weakly adhesive power, whitish	NICETACK removable tape NW (NICHIBAN Co. Ltd. Tokyo, Japan)
Cotton swab	To transfer a hair segment into a tube	Tapered, white color The tip should reach the bottom of a microtube	ASPURE industrial swab, AP-3 (AS ONE Corporation, Osaka, Japan)
Microtube	To enclose a hair segment	Capacity: 0.1- or 0.2-mL, single tube, flat cap, clear (8-strips, 12-strips, and dome caps are not recommended)	EU 0.1 mL single tube, low profile, BPK77201 (NIPPON Genetics Co. Ltd., Tokyo, Japan)
Magnifying glass	To see a hair segment carefully	Five-times or more powerful magnification A light is attached	LED stand loupe, SL-23 (AS ONE Corporation)
Tweezers	To attach a hair strand onto tape straight	Tapered enough to pick up a hair strand	Normal tweezers, thin stainless steel, IPT-02 (AS ONE Corporation)
White glove	To find a lost hair segment easily when it is on a hand	Thick nylon fiber (nitrile and latex gloves are not recommended because of sweatiness and strong grip performance)	Nylon gloves, EA354AM (ESCO Co. Ltd. Osaka, Japan)
White paper	To find a lost hair segment easily	Glossy	General white glossy paper, such as the reverse of poster, is available
Finger cot/glove	To press a hair strand onto tape	Disposable (white gloves described above are not applied to avoid contamination)	General disposable finger cots and gloves are available
Washing solvent	To wash a razor blade	Volatile, water-soluble solvent such as methanol	General analytical grade solvents are available
Adhesive swab	To catch a hair segment which is found on a place where it is difficult to collect with cotton swab	Comparatively strong adhesive power, clear or whitish	Peta swab, PS-2520 (ATOM KOUSAN Corporation, Tokyo, Japan)
Hood	To prevent wind and dust	Large enough to cover the workspace	Portable fume hood, 3–4064-33 (AS ONE Corporation)
Light stand	To light the workspace	Lighter the better	General products are available
Lint roller	To clean the workspace before and after segmentation procedure	White adhesive tape	General products are available
For drug extraction			
Tube rack	To array tubes containing a hair segment in turn	Compatible with 0.1-mL and 0.2-mL tubes, 96-well plate sized, with a removable lid It fits a microplate centrifuge rotor and a 12-channel pipette	96-Well preparation tray, 05–541-55 (Thermo Fisher Scientific Inc., Waltham, MA, USA)
Dot label	To label a tube	Diameter at 6.5 mm or less for 0.1- and 0.2-mL tubes	Standard dot label φ6.5 mm white, SDL-25–1 (Shamrock Labels, Bellwood, IL, USA)
Ultrasonic cleaner	To extract drugs from a hair segment	The chamber holds four or more tube racks The frequency and power of ultrasonic wave, and the chamber temperature are changeable	ASU-10D (AS ONE Corporation)
Twelve-channel pipette	To add the extraction solution in 12 tubes arranged in a row and transfer extracts into a microplate	Changeable (20–200 μL)	General products are available
Plate centrifuge	To sink a hair segment to the bottom of a tube	High speed as possible (over 1000 × *g*) Plates keep horizontal at stopping	PlateSpin (KUBOTA Corporation co., ltd., Tokyo, Japan)
Plate shaker	To mix extracts and diluents in microplates and 96-well plate-sized tube racks	Compatible with microplates	General products are available
Ninety-six well plate	To dilute extracts and inject analytical samples into an LC–MS/MS	Standardized by American National Standards Institute (ANSI), V-shaped bottom	PP plate 96 V, MS-3396P (Sumitomo Bakelite Co., Ltd. Tokyo, Japan)
Plate seal	To seal each well in a 96-well plate	Compatible with a 96-well plate It can be penetrated by the injector of an LC–MS/MS	RAPID EPS (BioChromato, Inc. Fujisawa, Japan)
Plate cover	To prevent dust into wells and onto a plate seal	Compatible to a 96-well plate	Plate common cover, P38C01N (Stem Corporation, Tokyo, Japan)
Airtight plastic container	To store an extraction solution and to keep a 12-channel pipette directly	It enables to store solutions for long time Wider than the width of a 12-channel pipette	Unix ware, TLO-10Ag (ASVEL Co. Ltd., Yamatokoriyama, Japan)

To stick a 0.4-mm segment to the cotton swab, the segment on the adhesive tape must be moved onto the grid paper because of the relatively strong adhesive power of the tape. The segment is slid toward the grid paper with the swab and rolled up onto the swab, as shown in Fig. 4a. The segments are carefully transferred from the swab surface to a microtube using a magnifying glass. The segment is placed near the bottom of a microtube by pushing the segment onto the inner wall of the tube while rotating the swab (Fig. 4b). The tube cap is closed, and it is confirmed under the glass that the segment is near the bottom of the tube. If the segment is on the upper wall of the tube, it should be knocked to the bottom by tapping.

If a segment is lost before placing it into a microtube, it should be thoroughly sought. A lost segment is typically found on grid paper, swabs, white gloves, or glass. To find a lost segment easily, the surfaces of the tools and workbench should be covered with white paper as completely as possible (Fig. 3b). Additionally, ‘walls’ should be made by folding and standing vertically on the front and back sides of the grid paper to minimize the likely search area of a lost segment (Fig. 4a). If a lost segment is found in a place where it is difficult to collect, an adhesive swab is used to catch the segment. The segment is transferred from the swab onto a double-sided adhesive tape and placed into a microtube using a tapered cotton swab.

Optional tools, such as a hood and light stand, as shown in Fig. 3b and Table 2, would also be helpful in the success of micro-segmentation.

### Drug extraction

Once a hair segment is placed into a tube, various drug extraction methods, used for general hair samples, are applicable. However, simple methods are suitable for MSA, because numerous analytical samples (typically 100 segments or more) must be prepared.

Kuwayama et al. [33–35] compared micropulverized extraction (MPE) with a stainless-steel bullet, liquid–liquid extraction (LLE) after alkaline dissolution [35–37], ultrasonication followed by soaking in an extraction solution (USE) [36, 37], and microwave-assisted extraction [38] using authentic hair strands, which were collected after chlorpheniramine (CP) ingestion [15]. The USE was less labor-intensive, although it took a long time (over 10 h) to extract sufficient analytes. The extraction rate of CP under the optimized USE conditions was almost the same as that in LLE and MPE. In USE, a mixture of the mobile phase consisting of an aqueous solution of 5 mM ammonium acetate and 0.05% formic acid (mobile phase A) and acetonitrile (3:1, v/v ) was used as the extraction solution. Next, the extraction solution (100 μL) containing CP-*d*_6_ (4 pg/mL) as the internal standard was added to the tube containing each 0.4-mm segment. The tube was sonicated at 23 kHz for 10 min, centrifuged, and maintained at approximately 22 °C in the dark for 24 h. The supernatant (35 μL) was transferred to a 96-well plate and diluted with mobile phase A (35 μL), and of the resultant solution, 50 μL was injected into an LC–MS/MS instrument.

The use of 0.1-mL or 0.2-mL microtube, 96-well tube rack, 96-well plate, and 12-channel pipette are effective to increase the throughput of sample preparation (Table 2). The 0.1-mL or 0.2-mL microtubes containing a segment were laterally aligned into a 96-well tube rack (Fig. 4c). A space of at least one row was left to simplify the following procedure. A dot label, where the tube identification (segment number, etc.) is written, is attached to the caps of the tubes aligned in a rack. The tube position should not be moved during the following procedure:

Typical components in the mobile phase used for general LC–MS/MS (water, acetonitrile, methanol, formic acid, acetic acid, and ammonium ion) are convenient extraction solutions because the extracts can be injected into an LC–MS/MS instrument without additional procedures, such as solvent evaporation and redissolution. The components of the extraction solution, frequency and power of the ultrasonic waves, sonication time, soaking time, and temperature can be adjusted for the efficient extraction of each analyte [39]. Generally, the recovery of analytes from hair segments in drug-containing regions increases with soaking time [26]. However, the main aim of MSA is to examine the position of specific drugs in the hair strand. Complete recovery of analytes is not always needed as long as a drug-containing region is found. Therefore, a reduction in extraction time can be prioritized over an increase in the amount of extracted analytes according to the analytical purpose.

### Instruments

In practical cases, MSA will be performed as a target analysis after it is confirmed that specific drugs are present in hair by conventional segmental analysis of a bulk hair sample. Instruments used for drug detection in MSA are required to possess the abilities of high speed to analyze many samples efficiently, and high sensitivity to quantify trace amounts of analytes in tiny hair segments. The selected reaction monitoring by a triple quadrupole LC–MS/MS instruments, which are produced as a comparatively high-end models by various MS instrument manufacturers, fulfills these requirements. Instrumental analysis is the rate-limiting step in several MSA procedures. LC instruments should have sample trays for multiple 96-well plates to automatically inject many samples. Under LC conditions, a high flow rate is preferable to achieve rapid analysis in less than 10 min per sample. In the setting of MS parameters, it is important to adjust the number of simultaneously monitored analytes and dwell times according to LC conditions.

In the analytical conditions adopted by Kuwayama et al. [15–17, 23–28], the time required for instrumental analysis was 7 min/sample, consisting of 2 min for large-volume injection and 5 min for MS measurement. Hence, sequential analysis of 192 samples in two 96-well plates, corresponding to a 7.68-cm hair strand, was completed in 22.4 h.

Quantification software provided by the instrument manufacturers in combination with Microsoft Excel is suitable for data analysis. The peak areas of the analytes were integrated automatically by quantification software. The numerical data were exported to the datasheet in Microsoft Excel, and drug concentrations were calculated. Distribution curves were generated by plotting drug concentrations per segment (Y-coordinate) versus segment numbers from the root to the tip of the hair strand (X-coordinate).

### Applications of MSA

MSA can be used to depict the high-resolution distribution of drugs on a hair strand. Even when one drug-containing region is observed using conventional segmental analysis, MSA may reveal that several peaks are present in the region. A high-resolution distribution provides two main pieces of information. The first is the precise distance from the proximal end to the peaks of the drug-containing region. The second is the peak shape of the drug-containing region. In the following sections, some examples of MSA applications are introduced.

### Examination of mechanism of drug uptake into hair

**Fig. 5:**
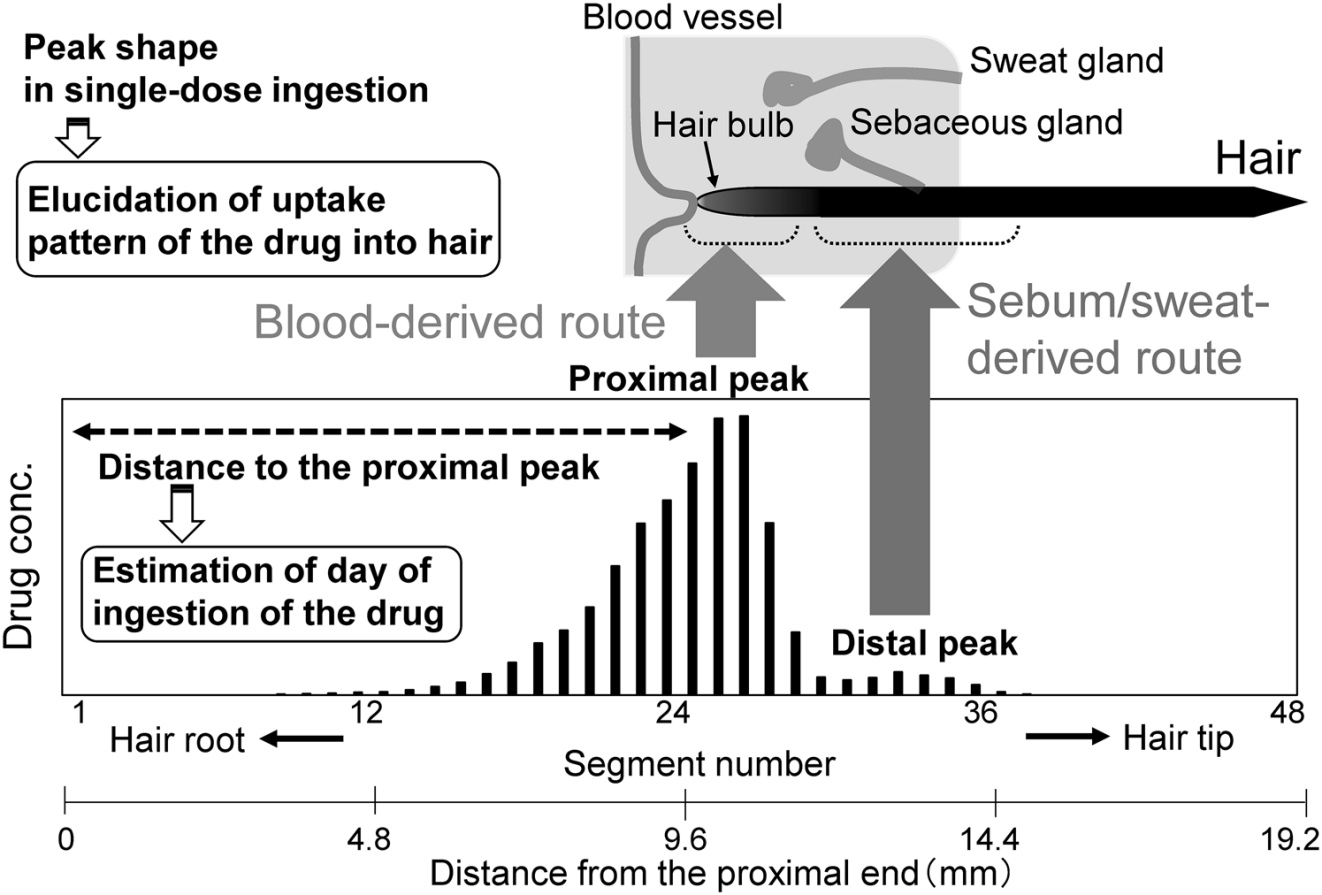
Drug distribution curve in a hair strand reflecting drug uptake routes

When a single hair strand collected several weeks after a single drug administration is analyzed using MSA, a distribution curve like that shown in Fig. 5 is typically obtained. The drug-containing regions cover over ten segments (4 mm), corresponding to a hair growth length of approximately 10 days. The peak shape consists of two peaks. The distance between the proximal and distal peaks is approximately eight segments, which is similar to the distance from the hair root end to the scalp (approximately 4 mm [40]). It is believed that ingested drugs can be taken up into the hair via two main routes [21, 22]. A drug in the capillary vessels surrounding the hair bulb can be taken up by the hair matrix cells. Second, a drug in sweat and sebum secreted from sweat glands and sebaceous glands, respectively, can soak into the hair region near the skin. Therefore, the proximal and distal peaks are considered to be derived from drug uptake via the blood and sweat/sebum, respectively, as shown in Fig. 5. Shima et al. [41] also examined the distribution of drugs in a single hair using segmental analysis at 1-mm intervals. These results supported the presence of proximal and distal peaks in the drug-containing hair region after a single drug administration.

**Fig. 6:**
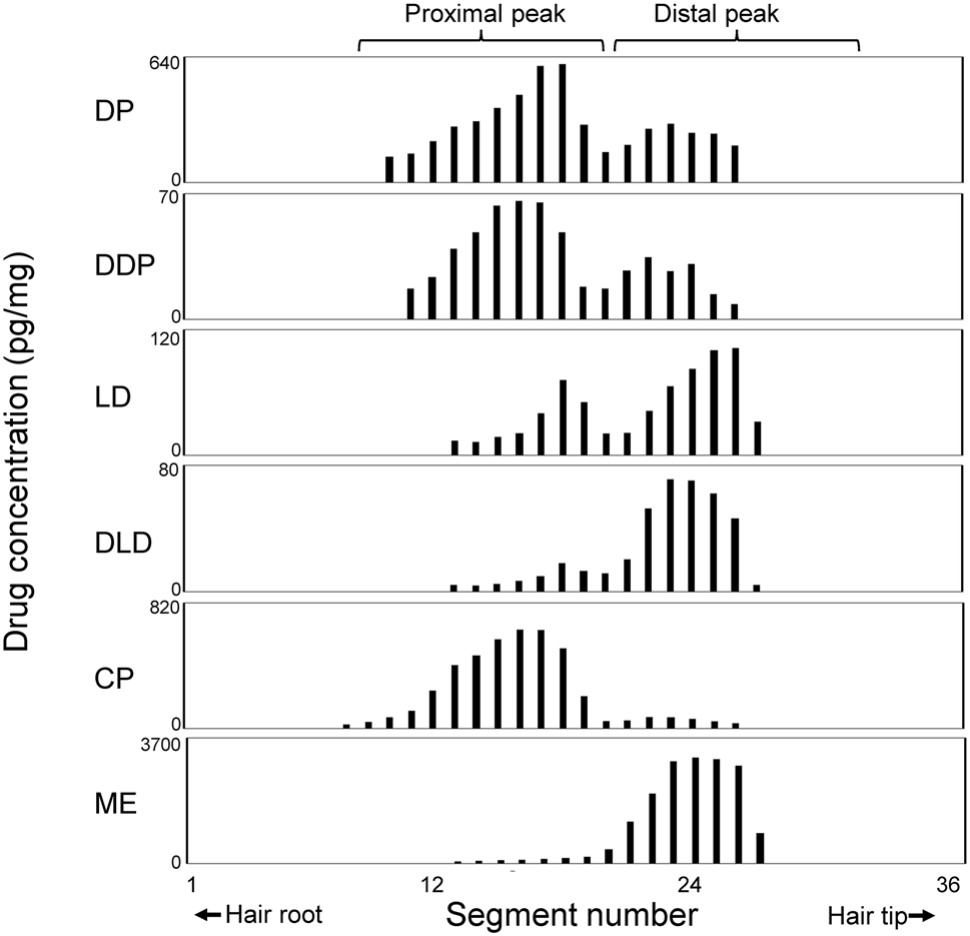
Different localizations of drugs administered simultaneously in a hair strand. An ointment containing diphenhydramine (DP) and lidocaine (LD) was applied to the skin of a participant, and he also orally ingested a tablet containing chlorpheniramine (CP) and methylephedrine (ME) on the same day.  A single hair strand was collected from the scalp several weeks after the drug administration. The figure quoted from [28] was modified for this figure. *DDP* desmethyldiphenhydramine, *DLD* desethyllidocaine

The proximal peaks were the major peaks for most analytes, which Kuwayama et al. [15–17, 23, 26, 28] previously examined using hair strands obtained after single administrations of various drugs. However, the distal peak was dominant for methylephedrine (ME) and lidocaine (LD) (Fig. 6) [16, 28]. Although the difference may be due to differential drug transfer into sweat and sebum depending on the chemical properties of the drugs and physiological states of tissues surrounding the scalp hair. Indeed, the correlation between the peak shape and chemical properties of each drug has not yet been elucidated. At present, it is difficult to determine the dominant route by which a target drug accumulates in the hair. The distribution of drugs whose peak shape has not been reported should be examined individually using a hair strand collected after ingestion of a single target drug. However, there are cases in which the peak shape of the target drug appears as one broad peak, such as the ME peak (Fig. 6), because the minor peak is very small. Therefore, the simultaneous ingestion of a target drug and a marker, such as CP, which is known to have two peaks, is recommended. The marker is useful for identifying whether a peak on the distribution curve of a target drug corresponds to a proximal (blood-derived) or distal (sweat/sebum-derived) peak. In the future, data on the peak shapes of many analytes will be accumulated, allowing the general mechanisms of drug uptake into hair to be elucidated.

### Estimation of the time of drug ingestion

One of the advantages of hair analysis is that it can estimate the time of drug ingestion based on the shift in the drug-containing region as a function of hair growth. In forensic cases, estimation of the day of drug ingestion using MSA would be effective in proving drug-facilitated crimes (DFCs) using hairs from victims rather than drug abusers (suspects) [25]. The distance between the proximal end and drug peak corresponds to the duration from drug intake to hair collection. If a hair constantly grows at 0.4 mm per day, the day of drug ingestion can be estimated using the segment number of drug peak on a distribution curve in a hair strand. However, the following points should be considered because estimations of the day by this method tend to include errors.

(1) Hair strands are normally cut near the scalp using scissors. The actual length of the hair remaining on the scalp is unknown, although it is estimated to be less than 1 cm (Fig. 1). The length must be considered when estimating the day of drug ingestion. If hair strands with the root are analyzed, the day of drug ingestion can be estimated more accurately using the distance from the hair root end.

(2) Hair strands in the telogen and catagen phases can be selected with a probability of approximately 15% [21, 22]. Reproducibility of the analytical results must be confirmed by analyzing two or preferably more hair strands independently.

(3) Individual hair strands grow at different rates even if they are collected from the same head region at the same time. This problem cannot be resolved unless the actual growth rate of the analyzed hair strands is measured.

**Fig. 7:**
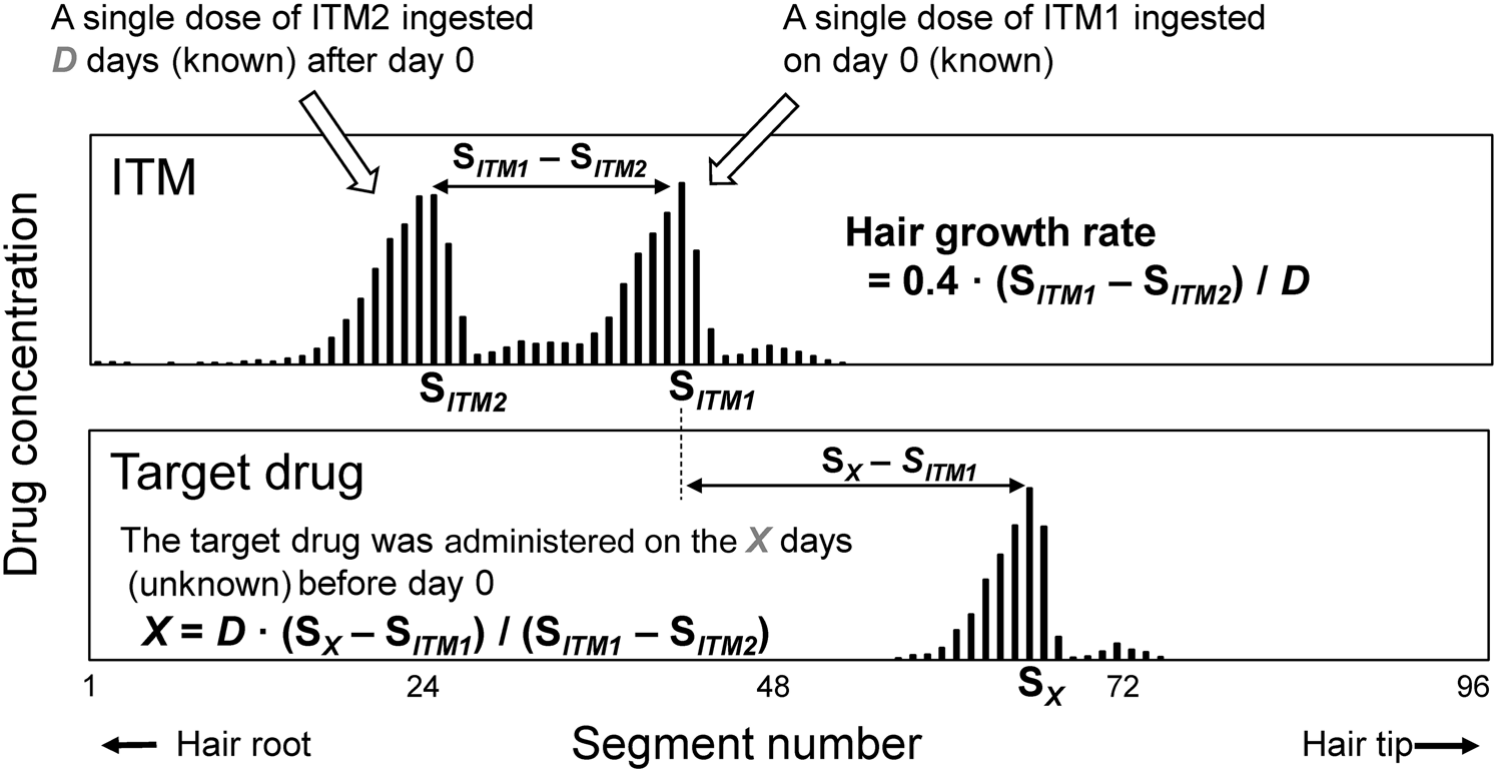
Accurate estimation of day of drug based on drug-containing positions in a hair strand using an internal temporal maker (ITM). *D* interval days between the administrations of ITM1 and ITM2, *S*_*ITM1*_*–S*_*ITM2*_ number of the hair segment between the ITM peaks

Kuwayama et al. [17, 23] devised a method to measure the actual growth rate of analyzed hair strands. They requested that participants take over-the-counter (OTC) medicines twice at an interval of several weeks before hair collection. Ingredients in OTC medicines, such as CP, were used as ITMs. The method used to accurately estimate the day of ingestion of a target drug using the ITM is shown in Fig. 7. First, the distribution curves of the ITM and the target drug were drawn. The two ITM peaks corresponded to the ITM ingested on two known days. The actual growth rate of the analyzed hair strands was measured by dividing the distance between ITM peaks by the interval days between the two ITM ingestions. Next, the distance between the target drug and ITM peaks was measured. The day of ingestion of the target drug was calculated using the distance and the actual hair growth rate. The accuracy of the estimation method was evaluated using various model drugs, such as fexofenadine, epinastine, and dihydrocodeine. The estimation errors of days for the target drugs decreased to within two days when using ITMs [17]. Kuwayama et al. [25] also applied this estimation method to an actual DFC case. In the DFC case, zolpidem was detected in the hair of the victim using conventional analysis. Subsequently, the victim ingested an OTC medicine containing CP twice at an interval of two weeks to cooperate with the investigation of DFC. MSA was performed to estimate the day of zolpidem ingestion using the ITM. The estimated day was consistent with the day of the incident that the victim remembered. The analytical results substantiated the victim’s statement objectively and contributed to clarifying the entire story of the DFC.

In the investigation of DFCs, ITM ingestion is useful to accurately estimate the time of drug ingestion. The required conditions for ITMs are: (1) the compound is not frequently ingested in daily life, (2) the compound is detected from a specific region of a hair strand reflecting the day of ingestion, (3) its use is not prohibited by laws, and 4) the ingestion does not adversely affect the health of participants. Basic compounds, such as CP, which are often present in OTC medicines, are suitable [17, 23, 25]. However, in terms of health and ethics, compounds with fewer pharmaceutical effects than OTC medicines are preferable. Ingredients in dietary supplements are potential alternative ITM candidates to OTC medicines. It is also expected that safer ITMs will be developed in the future.

If the participants have already ingested any prescribed medicines for their therapy that are known to be detectable in hair, these can be used as non-intentional ITMs [24]. The prescribed days and doses are recorded at the hospital and pharmacy. Additionally, it is unnecessary to request that the participants take any ITMs for research purposes. When estimating the incident day using MSA in the investigation of DFCs, it should be confirmed beforehand whether the participants (subjects and victims) had taken any prescribed medicines in the past.

Wiedfeld et al. [42] applied another approach to estimate the number of days of drug ingestion. They analyzed over 150 analytes using 2-mm segmentation of a single hair strand and estimated the mean growth rate of hair strands by comparing the hair positions of a target drug between strands collected on two different days. However, the estimated growth rate was not the actual growth one for the analyzed individual hair strands. ITMs ingested on known days are indispensable for accurately estimating the days of drug ingestion.

Recently, Xu et al. [43] applied MSA to drug-facilitated sexual assault cases. They showed the distribution of midazolam along the hair strands of the victim and demonstrated that MSA was useful in corroborating the narrative of the investigators in DFC cases.

MSA was also used to estimate the day of death [24]. A dead body was discovered and identified as being missing. Hair strands were plucked from the scalp of the corpse for toxicological examination. Upon background investigation, it was found that the person had been administered LD for surgery 57 days before the corpse was discovered. In this case, LD was used as a non-intentional ITM. The day of death was estimated based on the distance between the hair root end and LD peak.

### Visualization of drug-use history

**Fig. 8:**
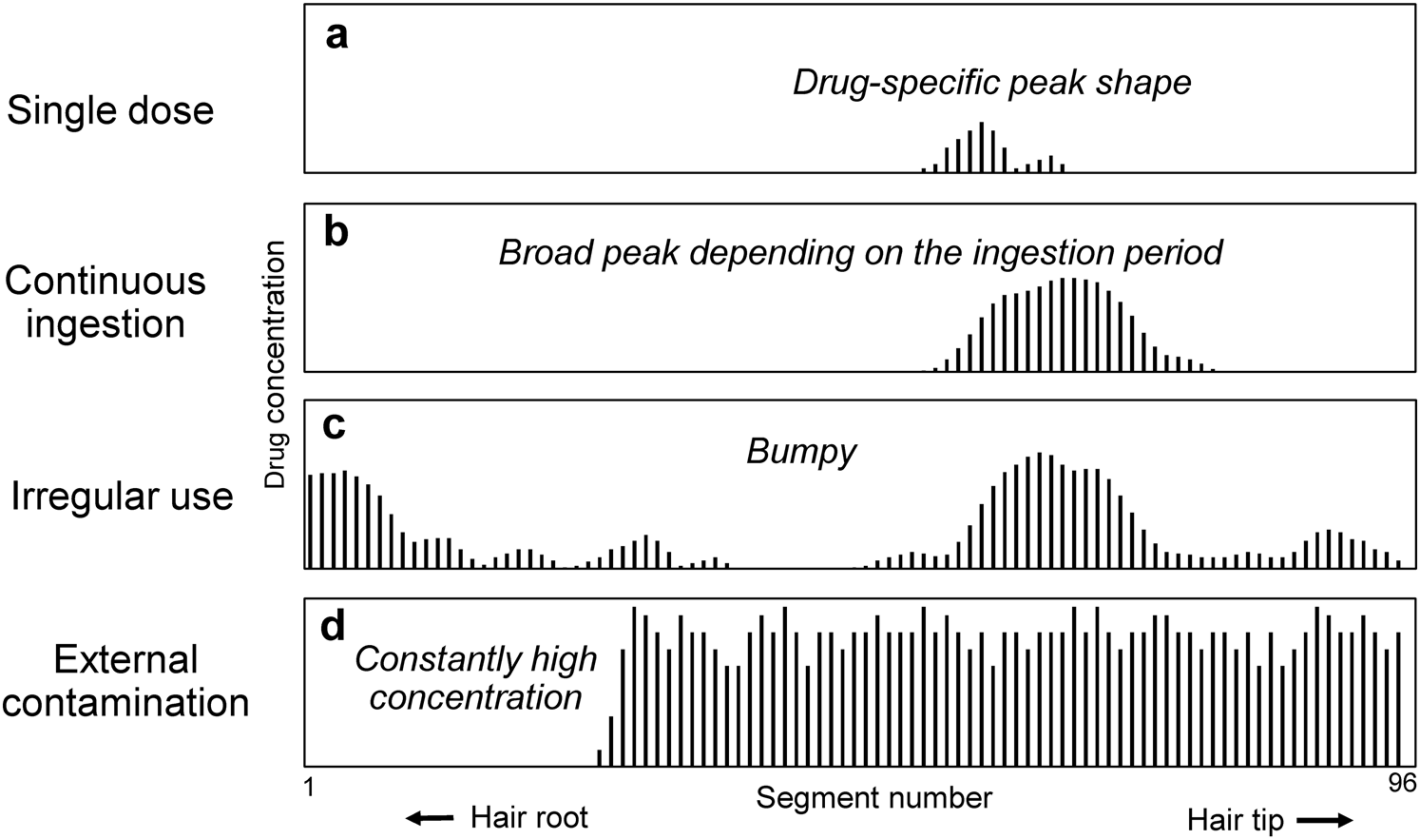
Differences of drug distribution curves in hair depending on drug-use history

A detailed drug distribution obtained by the MSA of an entire hair strand can reflect the history of drug use from the last habitual haircut to hair collection for drug testing. Four types of distribution patterns are observed according to the pattern of drug use (Fig. 8).

When a single dose of the drug is ingested, a peak shape characteristic of each analyte appears in a comparatively narrow region, as shown in Fig. 8a. In cases where a single dose of hypnotic is doped into food and drink in DFCs, this type of distribution curve would be typically observed for the victim’s hair strands.

When a drug is continuously ingested, as is the case for the regular administration of prescribed medicine, a broad peak can be observed (Fig. 8b). The peak width reflects the period of drug ingestion. However, it is difficult to determine the first and final days of drug ingestion because the positions of the start and end peaks in the distribution curve are obscure. The hair positions corresponding to the first and final days of drug ingestion were examined using hay fever medicines taken continuously for 1–18 days and ITMs taken as a single dose at intervals of several weeks [27]. The proximal and distal ends of the half-width of the broad peak were near the final and first days of drug ingestion, respectively. Thus, not only the day of single ingestion, but also the days of the first and final ingestion can be estimated accurately in the same manner as for a single dose of the drug. In practice, prescribed medicines that have already been administered continuously to the victims or suspects would be useful as non-intentional ITMs to estimate the day of intake of other drugs. Additionally, the method for estimating the days of continuous drug administration may be applicable for checking adherence to drug regimens using hair [44–46].

When a drug is taken irregularly, bumpy peaks appear, depending on the dose and time of drug intake (Fig. 8c). This type of distribution may be observed in the hair strands collected from drug abusers. As drug abusers usually take drugs depending on their mood and availability of the drug(s), the dose and time are uncertain. In addition, when suspects of drug abuse are arrested, they tend not to tell the truth about drug use. Such bumpy peaks can provide objective evidence of frequent drug use.

When a hair is externally contaminated with a drug, a high concentration of the drug is constantly detected along the hair on the distal side (Fig. 8d). The proximal end of the high-concentration region corresponds to the time at which the hair is in contact with the drug [28]. The drug is not detected in the proximal hair root region because the region has not yet been produced or was under the skin during the contact time. Drug concentration in contaminated hair is usually much higher than that during drug ingestion. Therefore, it would be easy to distinguish between ingested and adsorbed drugs externally by examining their distribution pattern and concentrations. If the metabolites characteristic of the target drug are detected, the distribution and concentration of the metabolites would also be effective indicators. Because external medicines, such as topical creams and sprays are often applied to the skin, the ingredients can adsorb onto the hair surface surrounding the affected area. Once the ingredients contact the hair surfaces and soak inside the hair strands, it is difficult to remove the contaminants using general hair washing procedures, such as sonication in aqueous solutions and organic solvents [28, 47–49]. It should be noted that ingredients in external medicines, such as diphenhydramine and LD, can contaminate hair during daily life.

## Cautious interpretation of distribution profiles using MSA

### Comparison among  hair strands

The activity of hair matrix cells differs among individual hair strands, even when they are present in the same head region of a person [21, 22, 50]. Therefore, it is not appropriate to compare the height of the drug peaks in the distribution curves between hair strands. However, a comparison of peak heights within a hair strand can reflect the doses of the drug ingested at the respective times. However, this interpretation is based on the assumption that the hair strand grows with constant cell activity at a constant rate. It must be confirmed that similar distribution results were obtained by analyzing two or more hair strands independently. If the distribution pattern in the second hair strand is significantly different from that in the first hair strand, the two hair strands would be in different phases of the hair cycle [21, 22, 40]. In such cases, MSA is repeated using different hair strands until a similar distribution profile is obtained. Unlike in bulk analysis, there is a low probability that the two analytical results match accidentally in MSA, because one distribution curve is a complex of analytical results from tens or hundreds of segments. Therefore, approximately five hair strands are collected for MSA, and at least two strands are analyzed.

### Hair damage

As the hair strand becomes longer, the drug use by the owner in the distant past can be revealed. However, hair surfaces can be damaged by washing, sunlight, and heat in daily life. Additionally, specialized hair treatments, such as bleaching, or dyeing, can significantly damage hair, which leads to decreased drug concentration in hair. Because the distal hair region of a long hair strand is produced early and is particularly exposed to external environments for a long time, the distal region would be damaged more than the proximal region. Therefore, when a distribution curve has some peaks for a specific drug, the peak present in the distal region may be underestimated in the interpretation of the dose. Kuwayama et al. [17] examined the variation in the height of CP peaks on a distribution curve after CP was taken at intervals of several weeks for 4 months. Although a significant decrease in the peak height in the distal region was not observed in hair strands without specialized hair treatments, exposure of hair to harsh environments could affect the drug distribution curve.

### Differences of hair properties

There are differences in the color and thickness of hair strands between races [21, 22]. In particular, pigments, such as melanin in hair strands, which determine hair color, can affect the uptake of drugs into the hair [51–54]. Miyaguchi et al. [51] examined the difference in zolpidem concentration between black and white hair strands using Japanese hair, because the previously reported concentration of zolpidem in hair after a single administration was inconsistent between French and Chinese hair strands [52, 53]. The concentration in Japanese black hairs was similar to that in Chinese hairs, but was much higher than that in white hairs. Shima et al. [55] examined the distribution of zolpidem and methoxyphenamine in black and white hairs. Their results suggested that hair pigments contribute to the incorporation of drugs into the hair around the hair root and retention of already incorporated drugs in the hair tissue. In any case, white hair would not be recommended as a specimen to investigate DFCs using MSA or conventional analysis. It is noteworthy that, at present, MSA has only been performed using hair strands from Asian adults and children (except for babies). Hair strands from races other than Asian and of all ages must be analyzed to finesse the interpretation of analytical results obtained using MSA and make them widely applicable to all humans.

Scalp hair is suitable for estimating the time of drug ingestion in terms of growth rate and percentage of hair strands in the anagen phase. However, pubic and axillary hair strands are not effective for estimating the time of drug intake, although they are sometimes used for drug testing as alternative hair specimens to scalp hair strands [56–59]. It is known that the pubic and axillary hair strands have a low percentage of hair strands in the anagen phase (40–60%) [21, 22]. Drugs were detected in only three hair strands out of twelve analyzed pubic and axillary hair strands [28]. In addition to the low percentage of hair strands in the anagen phase, the mechanism of drug uptake into the pubic and axillary hairs may be different from that of scalp hair.

In the past, experiments using the hair of animals, such as rats and pigs, have been performed as alternative experiments to human scalp hair [60, 61]. However, animal hairs are body hairs, and are greatly different from human scalp hair in terms of the drug uptake mechanism, hair cycle, and the percentage of hairs in the anagen phase. Therefore, it is impossible to extrapolate from the distribution results of animal hair to human hair using MSA.

## Limitations and perspectives of MSA

### Mechanization and automation of micro-segmentation

Micro-segmentation is not a difficult technique, although the careful work is labor-intensive. Practitioners must engage in the segmentation procedure several times. The time required to prepare a 0.4-mm segment (from cutting to 0.4 mm to collect the segment into a tube was approximately 20 s. That is to say, micro-segmentation of a 5-cm hair strand, which corresponds to a full travel distance in a slicer with a 50-mm scale, can be completed within 1 h. Because the manual collection of a segment is complicated, mechanization and automation technologies are expected to be developed. When Kuwayama et al. tested the motion to pick up a 0.4-mm segment and transfer it to a microtube using an electric micro-manipulator, it took much more time than manual preparation (unpublished observations). Additionally, manipulators cannot search for a lost segment when they fail to collect a segment and the segment flies away. Therefore, a mechanized and automated approaches are not employed at the present time.

### Further improvement of spatial resolution in hair segmentation

It is technically possible to cut a hair strand to a segment shorter than 0.4 mm (down to approximately 0.1 mm). However, the effectiveness would be low because a drug is distributed over a wide region (typically 4–8 mm) in a hair strand, even when a single dose of the drug is ingested [16, 27]. Although the spatial resolution of the distribution is improved by analyzing shorter hair segments, it takes much time and labor for the complicated segmentation procedure because the number of hair segments increases. Additionally, a more sensitive instrument may be required because the absolute amount of drug per segment decreases. Rather than shorter segmentation in the length direction, three-dimensional segmentation of a hair strand would be useful for examining the drug uptake mechanism. The radial drug distribution may provide more detailed information on the differences between the permeation of drugs in sweat and sebum on the hair surface and the incorporation of drugs in blood vessels into the hair matrix cells. However, preparing sequential sections in the radial direction would be difficult even using a microtome and a tissue slicer, as used for three-dimensional segmentation of nails [62], because the thickness of a hair strand is typically less than 0.1 mm [21, 22, 40]. Therefore, the development of a new technique for three-dimensional segmentation is required.

Kamata et al. [63] recently visualized drug distributions on a transverse section of a hair strand using MALDI-MS imaging and distinguished between hairs collected after a drug was ingested and those contaminated with a drug externally. The instruments for MALDI-MS imaging are very expensive, and their versatility is low as compared with the general LC–MS/MS instruments used for MSA. Additionally, specialized procedures, such as the preparation of thin hair sections and the application of matrix reagents, are more difficult and complicated than the procedure for MSA [9–11, 14, 63–71]. Therefore, MSA is easier to introduce as a new analytical method for drug testing in hair in many laboratories than MALDI-MS imaging. In any case, using both MSA and MALDI-MS imaging is the best method to elucidate the mechanism of drug uptake into the hair in fine detail.

### Improvement of MSA

MSA is essentially a target analysis because the number of ions monitored simultaneously is limited by shortening the measurement time per segment. The target drug is determined based on the results of non-target screening in the conventional hair analysis. High-resolution MS with an Orbitrap or time-of-flight mass analyzer is suitable for non-target analysis [25, 72–76]. However, no drug is sometimes found in practical cases, or else no drug is identified, although a small peak with ions characteristic of a specific compound is detected. To resolve this problem, Kuwayama et al. [26] developed a “selective concentration method” based on MSA. After a drug-containing region in a hair strand was identified using MSA, the remaining extracts from the region were concentrated in one tube and analyzed for drug identification using high-resolution MS. Although the selective concentration method is useful for the determination of drug-containing hair regions and drug identification, a combination of the conventional analysis and MSA using two types of MS is required. It consists of three steps: (1) non-target screening using high-resolution MS in the conventional analysis, (2) localization of a drug-containing hair region by MSA using triple quadrupole MS, and (3) identification of drug(s) in the concentrate using two different types of MS. An all-in-one MS instrument with high sensitivity, high speed, precise quantification for MSA, and high-resolution for drug identification has not yet been developed. If such an MS technique can be developed, it would be possible to perform drug screening, identify and quantify drugs, and determine the drug-containing hair region simultaneously in one step of MSA.

## Conclusions

This review summarizes the detailed procedures of MSA and its applications in forensic scenes so that many forensic laboratories can launch hair analyses using MSA. Most experimental tools and instruments used for MSA are those that general laboratories already possess or can purchase cheaply. Novice users would get accustomed to the procedures soon because MSA is not technically difficult. The analytical results provide powerful evidence of drug use in the investigation of drug-related crimes and detailed information about the mechanism of drug uptake into hair. In the future, the development of automatic segmentation instruments and improvement of MS instruments are expected to increase the effectiveness of MSA.
